# Effects of Non-invasive Brain Stimulation on Multiple System Atrophy: A Systematic Review

**DOI:** 10.3389/fnins.2021.771090

**Published:** 2021-12-13

**Authors:** Mengjie Zhang, Ting He, Quan Wang

**Affiliations:** ^1^Department of Occupational Therapy, Shanghai Yangzhi Rehabilitation Hospital (Shanghai Sunshine Rehabilitation Center), School of Medicine, Tongji University, Shanghai, China; ^2^Department of Rehabilitation Sciences, School of Medicine, Tongji University, Shanghai, China

**Keywords:** non-invasive brain stimulation (NIBS), repetitive transcranial magnetic stimulation, transcranial direct current stimulation, multiple system atrophy, motor function, cognitive function

## Abstract

**Background/Objective:** Multiple system atrophy (MSA) refers to a progressive neurodegenerative disease characterized by autonomic dysfunction, parkinsonism, cerebellar ataxia, as well as cognitive deficits. Non-invasive brain stimulation (NIBS) has recently served as a therapeutic technique for MSA by personalized stimulation. The primary aim of this systematic review is to assess the effects of NIBS on two subtypes of MSA: parkinsonian-type MSA (MSA-P) and cerebellar-type MSA (MSA-C).

**Methods:** A literature search for English articles was conducted from PubMed, Embase, Web of Science, Cochrane Library, CENTRAL, CINAHL, and PsycINFO up to August 2021. Original articles investigating the therapeutics application of NIBS in MSA were screened and analyzed by two independent reviewers. Moreover, a customized form was adopted to extract data, and the quality of articles was assessed based on the PEDro scale for clinical articles.

**Results:** On the whole, nine articles were included, i.e., five for repetitive transcranial magnetic stimulation (rTMS), two for transcranial direct current stimulation (tDCS), one for paired associative stimulation, with 123 patients recruited. The mentioned articles comprised three randomized controlled trials, two controlled trials, two non-controlled trials, and two case reports which assessed NIBS effects on motor function, cognitive function, and brain modulatory effects. The majority of articles demonstrated significant motor symptoms improvement and increased cerebellar activation in the short term after active rTMS. Furthermore, short-term and long-term effects on improvement of motor performance were significant for tDCS. As opposed to the mentioned, no significant change of motor cortical excitability was reported after paired associative stimulation.

**Conclusion:** NIBS can serve as a useful neurorehabilitation strategy to improve motor and cognitive function in MSA-P and MSA-C patients. However, further high-quality articles are required to examine the underlying mechanisms and standardized protocol of rTMS as well as its long-term effect. Furthermore, the effects of other NIBS subtypes on MSA still need further investigation.

## Background

Multiple system atrophy (MSA) refers to an adult-onset, fatal, progressive neurodegenerative disease characterized by autonomics dysfunction, parkinsonism, cerebellar ataxia, as well as cognitive deficits (Gilman et al., 2008; Fanciulli et al., 2019). The significant neuropathology characteristics exhibited by MSA contain degeneration of striatonigral and olivopontocerebellar structures, accompanied by distinctive glial cytoplasmic inclusions formed by fibrillated a-synuclein proteins (Koga and Dickson, 2018; Schweighauser et al., 2020).

MSA can fall into two main types either with parkinsonian-type MSA (MSA-P) or cerebellar-type MSA (MSA-C) based on the predominant clinical phenotype during assessment, and the predominant characteristic can vary over time (Gilman et al., 2008). MSA-P patients exhibit more parkinsonian signs (e.g., bradykinesia with rigidity, postural instability, tremor, and freezing of gait). In addition, the mentioned parkinsonian symptoms are observed in most MSA-C patients (Köllensperger et al., 2010; Low et al., 2015). The most frequent cerebellar characteristics in MSA-C patients are gait ataxia, accompanied by ataxic dysarthria and limb ataxia. The mentioned cerebellar signs are also present in around half of MSA-P patients (Köllensperger et al., 2010; Low et al., 2015).

Beyond the core clinical phenotype, autonomic failure (e.g., orthostatic hypotension, neurogenic lower urinary tract dysfunction, and constipation) and rapid eye movement sleep behavior disorder (RBD) are common pre-motor characteristics of MSA (Ito et al., 2006; Iodice et al., 2012; Figueroa et al., 2014; Lin et al., 2020). Besides, ~30% of MSA patients were diagnosed with several cognitive deficits, primarily as executive functions and verbal memory (Eschlböck et al., 2020).

However, there have been no effective treatments for MSA thus far. Existing pharmacological treatment of MSA is purely symptomatic (Rohrer et al., 2018; Mészáros et al., 2020). Only 30% of MSA patients benefit from levodopa therapy targeting parkinsonism, whereas the efficacy is limited and usually diminishes over time (Rohrer et al., 2018). Furthermore, long-term use of the mentioned medications often exerts adverse effects (e.g., hypotension, cognitive impairments, and hypersomnia) (Meissner et al., 2020).

Non-pharmacological approaches including physiotherapy and occupational therapy aim at improving symptoms and patient's quality of life. However, the evidence regarding physiotherapy or occupational therapy in MSA patients is limited (Jain et al., 2004; Raccagni et al., 2019; Coon and Ahlskog, 2021). Thus, non-invasive brain stimulation (NIBS) has recently become an alternative non-pharmacological therapeutic technique for MSA by personalized stimulation (Liu et al., 2018; Alexoudi et al., 2020). Transcranial magnetic stimulation (TMS) and transcranial direct current stimulation (tDCS) have been two extensively applied NIBS techniques.

TMS is a non-invasive, well-tolerated neurophysiological technique based on electro- magnetic induction. TMS has played a prominent role in the functional evaluation to characterize distinctive pattern of change at the final motor output stage between Parkinson's disease (PD) and atypical parkinsonian syndromes (e.g., MSA, progressive supranuclear palsy, and as well as corticobasal-ganglionic degeneration) (Kuhn et al., 2004). MSA patients showed abnormal motor cortex excitability upon TMS, with the reduction of short interval intracortical inhibition (SICI) following the increased motor thresholds and prolongation of ipsilateral and contralateral silent periods (Kuhn et al., 2004; Morita et al., 2008; Suppa et al., 2014). Moreover, inter-hemispheric inhibition measured by TMS could act as a possible neurophysiological correlate of cognitive dysfunction among MSA patients, since abnormal inter-hemispheric inhibition was correlated with cognitive impairment in MSA (Hara et al., 2018). Although the pathophysiological processes can be complex, the TMS articles suggest that dysfunction within the corticobasal ganglia-thalamocortical circuits form an important pathogenic basis for MSA. Additionally, intracortical facilitation and SICI have been shown significantly decreased in patients with RBD which suggests that through identifying subtle changes in the pathophysiology of the motor cortex, TMS can be a useful tool in the detection of very early stages of MSA (Lanza et al., 2020).

Several articles investigated the use of repetitive TMS (rTMS) for treating movement disorder [e.g., Parkinson's disease (PD), Tourette syndrome, dystonia, as well as essential tremor] (Lefaucheur et al., 2017; Tschöpe et al., 2021). The published data suggest that rTMS may mitigate motor symptoms in PD, whereas the evidence in other movement disorders remains unclear.

tDCS refers to another safe, cost-effective NIBS method, offering promise in mitigating motor impairment and improving cognitive and executive function in advanced PD (Lattari et al., 2017; Dagan et al., 2018; Lau et al., 2019). Given the possible regulatory effects of tDCS on cortical excitability, there have been numerous articles over the last decade applying tDCS technique for promoting cognitive and executive function (Doruk et al., 2014; Broeder et al., 2015; Manenti et al., 2018). Besides, existing articles have highlighted the therapeutic potential of tDCS in patients with cerebellar ataxias and the MSA-C (Ferrucci et al., 2016; Barretto et al., 2019; Chen et al., 2021).

There have been several systematic review and meta-analyses that investigating the effect of NIBS in Parkinson's disease and cerebellar ataxia (Goodwill et al., 2017; Kim et al., 2019; Chen et al., 2021). In the mentioned articles, MSA was regarded as one of atypical parkinsonism or degenerative ataxia. Up to present, there is no review has systematically assessed the effect of NIBS on MSA-P and MSA-C. This review hypothesized that NIBS can improve motor function and cognitive function in patients with MSA, and it can serve as an effective adjuvant therapy in MSA treatment. As a consequence, this systematic review aimed to summarize current interventions of NIBS in MSA-P and MSA-C and to examine the effects and safety of NIBS technique applied in the management of MSA-P and MSA-C.

## Methods

### Search Strategy

Seven electronic databases (e.g., PubMed, Embase, Cochrane Library, PsycINFO, CINAHL, CENTRAL, and Web of Science) were searched from inception to August 2021. The search strategy below was applied: (multiple system atrophy OR MSA-P OR MSA-C OR MSA) AND (transcranial magnetic stimulation OR TMS OR transcranial direct current stimulation OR tDCS OR transcranial alternating current stimulation OR tACS OR theta burst stimulation OR TBS OR non-invasive brain stimulation OR NIBS). Related systematic reviews and meta-analyses were identified, and the reference lists of them were checked. Two authors (MZ and TH) identified the potential articles independently by complying with the uniform screening criteria and any disagreements were settled through discussion.

### Eligibility Criteria

Articles meeting the criteria were included: adult participants diagnosed with MSA (e.g., probable MSA or possible MSA) in accordance with the clinically diagnostic criteria (Gilman et al., 2008); interventions were NIBS (e.g., TMS or tDCS), and NIBS was employed for therapeutic purposes; outcomes of interest consisted of symptoms, motor function, cognitive function and brain modulatory effects and others; controlled or exploratory articles; peer-reviewed articles and published in English. Articles were excluded if: NIBS was intended for assessing neurophysiological measures; conference papers, abstracts and other articles whose full text is not available; reviews, editorials, commentaries, and other non-clinical trials.

### Study Selection

First, the Endnote software was adopted to remove duplications after completing the search process. Second, the author screened relevant articles through reading titles and abstracts. Third, the full-text of the remaining articles were read for in-depth screening.

### Data Extraction and Methodological Quality Assessment

A customized form was adopted to collect data of the included articles. The data (i.e., study characteristics, characteristics of study subjects, intervention details, and outcome measures of included articles) were extracted. The Physiotherapy Evidence Database (PEDro) scale was used to assess the methodological quality of included articles (de Morton, 2009). By complying with this criterion, those with a score below three were classified as low quality, while those with a score between 4 and 6 were classified as moderate quality, and those with score above seven were classified as high quality. Two independent reviewers conducted the data extraction and quality assessment. Any discrepancies were resolved through face-to-face discussions.

## Results

### Study Selection

**Figure 1:**
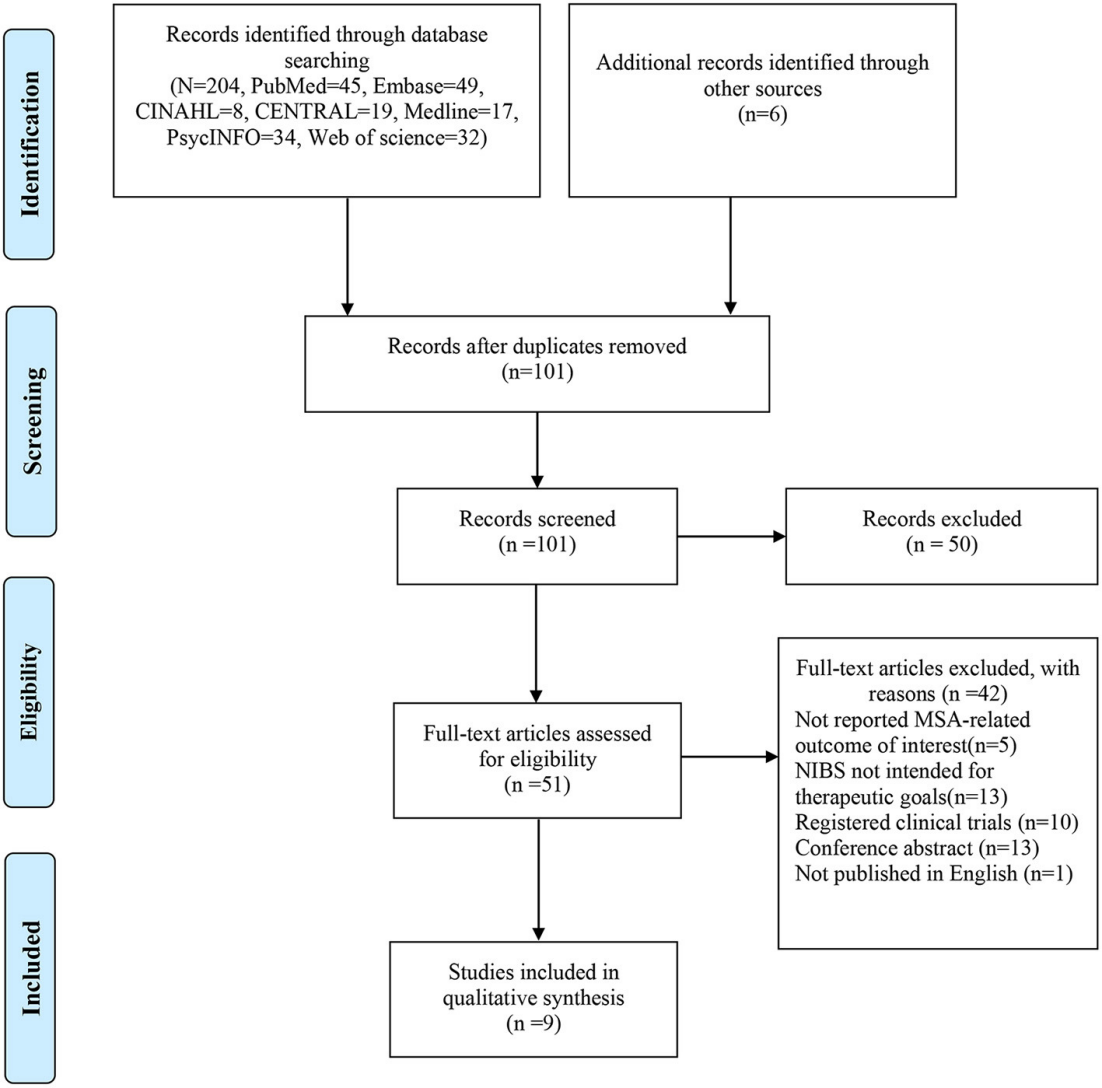
Study selection process.

The study selection is illustrated in Figure 1. Initially, 210 records were identified by searching electronic databases and hand searching. After removing duplications, 101 records were retained, of which 50 records were excluded after screening the titles and abstracts. Of the other 51 articles, 42 were removed after reading the full text. Lastly, 9 articles were eligible for qualitative analysis.

### Methodological Quality of Reviewed Articles

The results of methodological quality assessment of the included articles were presented in Table 1. Nine included articles were published between 2013 and 2020, of which three were randomized controlled trials (Benussi et al., 2015; Chou et al., 2015; Wang et al., 2016; Song et al., 2020), two were controlled trials (Kawashima et al., 2013; Yildiz et al., 2018), two were case report (Wang et al., 2017; Alexoudi et al., 2020) and one was non-controlled trial (Liu et al., 2018).

**Table 1:** The PEDro scale scores of included studies.

**References**	**Eligibility criteria**	**Random allocation**	**Concealed allocation**	**Baseline comparability**	**Blind subjects**	**Blind therapists**	**Blind assessors**	**Adequate follow-up**	**Intention-to-treat analysis**	**Between-group comparisons**	**Point estimates and variability**	**Scores**
Kawashima et al. (2013)	✓	×	×	✓	×	×	×	✓	✓	✓	✓	5
Benussi et al. (2015)	✓	✓	×	✓	✓	×	✓	✓	✓	✓	✓	8
Chou et al. (2015)	✓	✓	×	✓	✓	×	✓	✓	×	✓	✓	7
Wang et al. (2016)	✓	✓	×	✓	✓	×	×	✓	✓	✓	✓	6
Liu et al. (2018)	✓	×	×	×	×	×	×	✓	✓	✓	✓	4
Yildiz et al. (2018)	✓	×	×	×	×	×	×	✓	✓	✓	✓	4
Song et al. (2020)	✓	✓	×	✓	✓	×	✓	✓	✓	✓	✓	8

Except for two case reports, only three articles were classified as high quality (Benussi et al., 2015; Chou et al., 2015; Song et al., 2020), and four were moderate quality (Kawashima et al., 2013; Wang et al., 2016; Liu et al., 2018; Yildiz et al., 2018). All articles did not describe allocation concealment, and only three articles (Benussi et al., 2015; Chou et al., 2015; Song et al., 2020) blinded subjects and therapists. Regarding the comparison on baseline characteristics, five articles (Kawashima et al., 2013; Benussi et al., 2015; Chou et al., 2015; Wang et al., 2016; Song et al., 2020) reported a comparable level between the intervention group and the control group. Besides, intention to treat analysis was performed in six articles (Kawashima et al., 2013; Benussi et al., 2015; Wang et al., 2016; Liu et al., 2018; Yildiz et al., 2018; Song et al., 2020).

### Participants

The demographic characteristics of the included articles were presented in Table 2. The number of participants was 123 in total, and the number of sample sizes ranged from 1 to 50. The mean age of participants ranged from 52.71 (Wang et al., 2016) to 67.8 (Benussi et al., 2015) years. All articles, except for the two case reports, included participants of both genders, with a higher proportion of men overall. The mean duration of symptoms ranged from 2.18 (Wang et al., 2016) to 5.7 (Benussi et al., 2015) years. Only three articles (Chou et al., 2015; Wang et al., 2016; Alexoudi et al., 2020) used the Hoehn and Yahr scale (H&Y) to describe the severity of recruited samples, and the mean of H&Y scores ranged from 3.2 to 4.

**Table 2:** Clinical and demographic characteristics of the patients and technical aspects of the reviewed studies.

**References**	**Design**	***N***, **G (F/M)**	**Age (y)**	**DD (y)**	**Severity (HY)**	**Intervention**	**Outcome measures**	**Main results**
**NIBS in MSA-P**								
Kawashima et al. (2013)	Controlled	MSA-P: 10 (6/4) PD: 10 (6:4)	MSA-P: 59.5 (11.2) PD: 66 (7.7)	MSA-P: 2.76 (0.9) PD: 2.76 (0.7)	NR	PAS Stimulation site: right median nerve (ES); left M1 (TMS) Key parameters ES: Intensity = 110% RMT; TMS: Intensity = SI1 mV; Frequency = 0.2 Hz; Total = 240 pairs of stimuli; Duration = 20 min with an interstimulus interval of 25 ms; F8c; single session	UPDRS; MEP	No change in the averaged amplitude of MEPs
Chou et al. (2015)	RCT, double blinded	Active: 9(3/6) Sham:10(6/4)	Active: 55 (7) Sham: 54 (2)	Active: 2.5 (1.58) Sham: 2 (1)	Active: 3.2 (0.9) Sham: 3.2 (0.7)	rTMS Stimulation site: left M1 Key parameters Frequency = 5 Hz Intensity = 110% RMT 10 trains of 100 pulses with an intertrain interval of 40 s)/session; F8c;10 sessions	UMSARS- II and resting-state functional connectivity	Significant rTMS-related changes in motor symptoms and functional connectivity in active rTMS group
Wang et al. (2016)	RCT, single blinded	MSA-P: 15 (8/7) HC: 18 (9/9)	MSA-P: 53.40 (4.69) HC: 55.17 (3.20)	MSA-P: 2.18 (1.33)	MSA-P: 3.23 (0.70)	rTMS Stimulation site: left M1 Key parameters: Frequency = 5 Hz Intensity = 110% RMT 10 trains of 100 pulses/session F8c;10 sessions	UMSARS- II	Significant decreased UMSARS-II scores in active rTMS group
Wang et al. (2017)	Case report	1, Female	61	4	NR	rTMS Stimulation site: left M1 Key parameters: Frequency = 5 Hz Intensity = 110% RMT 10 trains of 100 pulses/session F8c;10 sessions	UPDRS-III; CMCT	Significant improvement in UPDRS-III and specific task performance; shortened CMCT
Alexoudi et al. (2020)	Case report	1, Female	66	5	4	tDCS Stimulation site: motor and pre-motor cortices (anodal), mastoids (cathodal) Key parameters: 2 mA; 30 min 10 sessions	UPDRS III, TUG; RAVLT; DSST-WAIS-III, TMT-A	Improvement in UPDRS III and the TUG test; positive effect in RAVLT, the DSST-WAIS-III and the TMT-A
**NIBS in MSA-C**								
Benussi et al. (2015)	RCT, double blinded	4/2	67.8 (8.3)	5.7(2.7)	NR	tDCS Stimulation site: cerebellum (anodal), right deltoid muscle (cathodal) Key parameters: 2 mA; 20 min single session	SARA; ICARS; 8MW; 9HPT	Significant improvement in SARA, ICARS, 8MW and 9HPT.
Yildiz et al. (2018)	Controlled	MSA-C: (12, 4/8) AD: (5, 2/3) HC: (9, 4/5)	MSA-C: 56.7 (6.9) AD: 80 (4.2) HC: 53.4 (7.7)	MSA-C: 2.94 (1.5) AD: 2.4 (0.49)	NR	rTMS Stimulation site: cerebellum Key parameters: Frequency = 1 Hz Intensity = 90% RMT 600 pulses/session F8c; single session	SAI Reaction Time	SAI responses got improved in MSA-C group
Song et al. (2020)	RCT, double blinded	Active: 25 (11/14) Sham: 25 (10/15)	Active: 53.1 (8.1) Sham: 53.2 ± 9.4	Active: 2.7 (1.1) Sham: 2.5 (0.9)	NR	rTMS Stimulation site: cerebellum Key parameters: iTBS Frequency = 50 Hz Intensity = 80% RMT Total pulse = 1,800 F8c;10 sessions	Dynamic cerebello- fronto connectivity; SARA	Improvement of cerebello-frontal connectivity and balance functions
**NIBS in MSA-P and MSA-C**								
Liu et al. (2018)	Non-controlled	9 (5/4)	58.0 (7.0)	2.39 (0.78)	NR	Stimulation site: cerebellum AND bilateral M1 Key parameters: Frequency = 5 Hz Intensity = 100% RMT 2,000 pulses and 50 trains Round coil; 5 sessions	UMSARS-II; resting-state brain activity	Increased motor network resting-state complexity

*Nr, number; F, female; M, male; DD, disease duration; rTMS, repetitive transcranial magnetic stimulation; MSA-P, Multiple system atrophy- predominant Parkinsonism; MSA-C, Multiple system atrophy-predominant cerebellar ataxia; PD, Parkinson's disease; NR, not reported; F8c, figure eight-shaped coil; ES, electric stimulation; TMS, transcranial magnetic stimulation; M1, primary motor cortex; PAS, Paired Associative Stimulation; RMT, resting motor threshold; UMSARS, Unified Multiple System Atrophy Rating Scale; MEP, motor-evoked potential; RCT, randomized controlled trial; HY, Hoehn and Yahr scale; HC, healthy control group; CMCT, Central motor conduction time; SAI, short-latency afferent inhibition; iTBS, intermittent theta burst stimulation; SARA, Scale for Assessment and Rating of Ataxia scores; ICARS, International Cooperative Ataxia Rating Scale; 8MW, 8-Meter Walking Time; 9HPT, Nine-Hole Peg Test; TUG, Timed Up and Go test; RAVLT, Rey's Auditory Verbal Learning Test; DSST-WAIS-III, Digit Symbol Substitution Test-Wechsler Adult Intelligence; TMT-A, Trail Making Test*.

### Intervention Characteristics

The characteristics of interventions of the included studies were shown in Table 2.

Among the nine articles explored the therapeutic effect of NIBS on MSA, rTMS was applied in six articles (Chou et al., 2015; Wang et al., 2016, 2017; Liu et al., 2018; Yildiz et al., 2018; Song et al., 2020), and tDCS was applied in two studies (Benussi et al., 2015; Alexoudi et al., 2020), the paired associative stimulation (PAS) was used in the remaining one (Kawashima et al., 2013). Stimulation site was left primary motor cortex (M1) in four articles (Kawashima et al., 2013; Chou et al., 2015; Wang et al., 2016, 2017), cerebellum in two articles (Yildiz et al., 2018; Song et al., 2020), and one study simulated both cerebellum and bilateral M1 (Liu et al., 2018). High frequency stimulation ranged from 5 to 50 hz was performed in five articles (Chou et al., 2015; Wang et al., 2016, 2017; Liu et al., 2018; Song et al., 2020), and low frequency stimulation between 0.2 and 1 hz was performed in two articles (Kawashima et al., 2013; Yildiz et al., 2018). The duration of intervention varied from single session to 2 weeks. Only one study reported follow up period, which was 3 months after first stimulation (Alexoudi et al., 2020).

### Outcomes

Description of outcomes of the included articles was grouped by complying with the NIBS employed and the subtypes of MSA.

### NIBS in MSA-P

There were five articles explored the effects of NIBS in MSA-P patients.

### rTMS Articles

Three articles employed rTMS in MSA-P. Chou et al. (2015) performed a randomized sham-controlled study on 19 MSA-P patients using 10 sessions of high frequency (5 hz) rTMS over the left M1 and reported a significant improvement of motor symptoms, as measured by UMSARS-II, in comparison with sham stimulation (Chou et al., 2015). The authors exploited functional magnetic resonance imaging (fMRI) to measure brain resting-state functional connectivity. They found that positive changes in functional connectivity of functional links involving the DMN, cerebellar network, and limbic network were only identified in the active rTMS group. Moreover, the amelioration of motor symptoms was correlated with positive changes in functional connectivity after active rTMS stimulation.

Wang et al. (2016, 2017) adopted an intervention protocol that was almost identical to Chou et al. (2015). The case report of Wang et al. (2017) showed a significant improvement in UPDRS-III and finger tapping, hand alternating, and heel tapping performance (Wang et al., 2017). Additionally, central motor conduct time of both sides were shortened after rTMS stimulation in comparison with baseline.

Likewise, Wang et al. (2016) conducted a randomized-sham controlled study on 15 MSA-P patients and reported improved motor function and promoted activation of bilateral cerebellum in the real rTMS group other than the sham one (Wang et al., 2016). However, different from the finding of Chou et al. (2015), no correlation was identified between improvement of motor function and increase of cerebellum activation.

### PAS Article

Kawashima et al. (2013) employed a single session of low frequency (0.2 hz) TMS over left M1 and electrical stimulation in the right median nerve at the wrist in 10 MSA-P patients. The variations of motor-evoked potential (MEP) amplitudes were determined to reveal the effect of PAS on motor cortex excitability. Furthermore, no significant difference was reported in M1 function before and after stimulation (Kawashima et al., 2013).

### tDCS Article

Alexoudi et al. (2020) employed 10 sessions (30 min per session) of anodal tDCS over motor and pre-motor cortices in a 66-year-old woman. Improvements were reported in motor function after tDCS, as well as in activities of daily living, visuomotor activity and processing speed, and working memory. Importantly, the treatment effect lasted for 3 months (Alexoudi et al., 2020).

### NIBS in MSA-C

There were three articles explored the effects of NIBS in MSA-C patients.

### rTMS Articles

In the randomized controlled trial conducted by Song et al. (2020), the effects of 10 sessions of high frequency (50 hz) intermittent theta-burst stimulation (iTBS) on bilateral cerebellum in 50 MSA-C patients were examined. According to the results, in the active iTBS group, a significant of improvement of motor imbalance and cerebello-frontal connectivity was reported, as revealed by the Scale for Assessment and Rating of Ataxia (SARA) scores and TMS-EEG, respectively. Furthermore, the SARA scores were significantly and negatively correlated with the neural activity of frontal connectivity from 80 to 100 ms after iTBS intervention (Song et al., 2020).

Different from the above articles, Yildiz et al. (2018) performed a controlled clinical trial using single session of low frequency (1 hz) rTMS stimulation over cerebellum in 12 MSA-C patients and found impaired short-latency afferent inhibition (SAI), attention and the spatial working memory, as measured by reaction time, were significantly improved, in comparison with baseline (Yildiz et al., 2018).

### tDCS Article

Benussi et al. (2015) performed a randomized sham-controlled trial to examine the effect of a single session cerebellar anodal tDCS that lasts 20 min in six MSA-C patients. The authors reported significant improvement in severity of ataxia, finger dexterity and upper limb coordination, as assessed by SARA, International cooperative ataxia rating scale (ICARS) and the nine-hole peg test (9HPT), respectively, in real tDCS group, in comparison with sham one. However, no significant difference was identified in gait speed, as measured by the 8-Meter Walking Time (8MW), between the two groups (Benussi et al., 2015).

### NIBS in MSA-P and MSA-C

Liu et al. (2018) performed a non-controlled trail to explore the effects of high-frequency rTMS over bilateral M1 and cerebellum in three MSA-P patients and six MSA-C patients. Although only five sessions stimulation were applied, the authors found a significant improvement in motor function and an increase in resting-state complexity within the motor network, as recorded by UMSARS-II and blood-oxygen-level dependency (BOLD) functional magnetic resonance imaging, separately. Consistent with previously achieved results, the improvement in motor function was positively correlated with the increase in motor network resting-state complexity. However, the authors did not report the efficacy of rTMS in MSA-P and MSA-C respectively by complying with disease types (Liu et al., 2018).

## Discussion

To the best of the author's knowledge, this review has been the first review that systematically investigated the existing evidence of the use of NIBS (e.g., rTMS, tDCS, and PAS) for treating MSA-P and MSA-C. The recruited articles were reported with significant heterogeneity and variability of study population, study designs and interventional protocols, thereby increasing difficulty in drawing a definite conclusion of the prospect of NIBS techniques. However, most of the included articles reported improving effects of NIBS on the motor function, cognitive function and cortical function of MSA patients. As impacted by the low number of included articles and the low quality of articles, there is no sufficient confidence in mentioned findings, and the estimates should be interpreted cautiously.

Over the past few years, the effects of NIBS on MSA have been increasingly studied, whereas the exact mechanisms remain unclear. rTMS uses repeated magnetic pulses *via* a stimulation coil placed over the scalp to generate electromagnetic fields that are capable of inducing action potential in the brain (Valero-Cabré et al., 2017). To be specific, cortical excitability is enhanced by high-frequency rTMS (≥5 Hz), while it is inhibited by low-frequency rTMS (≤1 Hz) (Pascual-Leone et al., 1994). tDCS delivers weak direct currents to the cortex *via* two electrodes attached to the scalp. The stimulation consists of two types, i.e., anodal stimulation excites neuronal activity and while cathodal stimulation can suppress neuronal activity. rTMS and tDCS regulate cortex excitability in different manners. Specifically, rTMS can induce direct and trans-synaptic neuronal activation, while tDCS can lead to subthreshold neuronal membrane polarization. However, the long-term potentiation or depression (LTP/LTD)-like synaptic plasticity was found to be induced by both methods (Nitsche et al., 2003; Esser et al., 2006; Monte-Silva et al., 2013).

According to all the included articles examining the role of NIBS in treating MSA-P, the targeted area of stimulation was the M1. Nevertheless, in the MSA-C articles, the cerebellum was selected for stimulation. Existing articles exploited theta-burst stimulation to examine M1 excitability in MSA and reported reduced short-interval intracortical facilitation in MSA-P and MSA-C patients, thereby demonstrating impaired M1 plasticity (Suppa et al., 2014). Moreover, in the longitudinal study conducted by Burciu et al. (2016), the authors reported decreased functional activity presented by task-related fMRI signal in M1, supplementary motor area and superior cerebellum in MSA over a 1-year period. All the mentioned revealed that the role of brain plasticity is of pivotal importance in the treatment of MSA.

Motor impairments in MSA are considered to be attributed to dysfunction of the cerebellum and the neural networks it connects to Lu et al. (2013). Through the cerebello-thalamo-cortical circuit, the cerebellum and the bilateral M1, a connected network of the cerebellum, are critical to motor control (Grimaldi et al., 2014). Pukinje cells within the cerebellum have physiological inhibitory effects on the M1 by inhibiting the dentate nucleus (Spampinato et al., 2020), which is termed cerebellar brain inhibition (CBI) (Galea et al., 2009) MSA impairs the regulation of the dentate nucleus and Purkinje cells, thereby decreasing the excitability of M1 and ultimately leading to motor control dysfunction (Yang et al., 2019a).

Liu et al. (2018) applied rTMS over cerebellum and bilateral M1, and the beneficial effect of rTMS might be correlated with the direct activation of M1 and the reduction of M1 inhibition by the cerebellum (Liu et al., 2018). Since the cerebellum and M1 are functionally connected, in-depth research should be conducted to verify whether stimulating the two regions is better than targeting either region separately.

In the included articles of high-frequency rTMS in MSA-P, its regulatory effect on the brain was found, which was manifested as the improved default mode network (DMN) plasticity and cerebellar activation. The underlying mechanisms of DMN modulation remain unclear, DMN plasticity may show sensitivity to rTMS treatment and facilitate the consolidation and maintenance of brain function *via* DMN plasticity (Fjell et al., 2014; Chou et al., 2015). Wang et al. (2016) hypothesized that the increase in cerebellar activation was correlated with the motor effect in MSA, which is probably attributed due to cerebellar loop compensation induced by high-frequency rTMS treatment. DMN is closely correlated with the cerebellar and limbic networks (Catani et al., 2013; Halko et al., 2014). DMN exhibits the maximal activation during rest, which is correlated with a high degree of neuroplasticity (Shulman et al., 1997; Fjell et al., 2014). Accordingly, it can be speculated that rTMS may change the excitability of the motor cortex by regulating the brain plasticity in MSA patients.

Furthermore, central motor conduction time (CMCT) was reported to be prolonged in MSA patients (Abbruzzese et al., 1997). CMCT is the time it takes for nerve impulses to reach the target muscles based on the central nervous system. Since the CMCT decreased after the high-frequency rTMS treatment, the improvement in trans-synaptic efficiency might be reflected (Wang et al., 2017).

Inconsistent with high-frequency rTMS, low-frequency rTMS can temporarily inhibit cortical excitability (Kobayashi and Pascual-Leone, 2003). Articles reported that low-frequency rTMS on the lateral cerebellum impacted the excitability of the motor cortex for 30 min (Chen et al., 1997; Heide et al., 2006). As indicated from articles, rTMS targeting the cerebellum could inhibit the excitability of Purkinje cells, thereby inhibiting the dentate nucleus and in turn the contralateral M1 (Ugawa et al., 1995). Besides, the possible mechanism underlying the therapeutic benefits of rTMS was found as the neuroprotective effect of rTMS. According to May et al. (2007), the gray matter volume at the left superior temporal gyrus increased significantly after 1 Hz rTMS was applied for 5 days in the identical site (May et al., 2007). Cerebral cortex and cerebellum atrophy is evident in MSA-P and MSA-C. Yang et al. (2019b) identified gray matter loss in anterior and posterior cerebellar lobes. Moreover, they found the negative correlation between the extent of atrophy involving left lobule IX and motor performance. Next, they observed a negative correlation between the extent of cerebellar volume loss and cognitive impairments (Yang et al., 2019b), and the mentioned findings comply with those of others (Kim et al., 2015). It can be therefore speculated that rTMS may increase gray matter of cerebellum, which can improve motor and cognitive function in MSA patients.

Likewise, the exact mechanisms of cognitive impairment in MSA remain unclear. As demonstrated from neuroimaging, neuropsychological and neuropathologic articles the cognitive decline in MSA may originate from the atrophy of the cerebral cortex (especially the frontal lobes), the subcortical structure, as well as the cerebellum (Stankovic et al., 2014; Lee et al., 2016; Barcelos et al., 2018; Santangelo et al., 2018; Caso et al., 2020). To be specific, the lesions of subcortical circuit loop (i.e., the cerebral cortex-basal gangliathalamus-cerebral cortex circuit and the cerebral cortex-pons-cerebellumthalamus-cerebral cortical circuit) may cause essential signals conduction impairment (Miyachi, 2009; Zhang et al., 2018).

Although cognitive deficits are significantly common in MSA patients, only one of the recruited articles examined the effects of rTMS on the treatment of cognitive impairment (Yildiz et al., 2018). The effects of rTMS on cognitive enhancement have been reported in mild cognitive impairment and Alzheimer's disease. Executive performance can be significantly improved by high-frequency rTMS over the right inferior frontal gyrus, and memory functions can be noticeably improved by low-frequency rTMS targeting on the left dorsolateral prefrontal cortex (DLPFC) (Chou et al., 2020). According to Minnerop et al., significant hypoperfusion was reported in the frontal and dorsolateral prefrontal cortex and in MSA-P patients and the severity of cognitive impairment correlated with hypoperfusion in the DLPFC. Compared with MSA-P, visuospatial cognitive and construction impairment was more significant in MSA-C patients, and it was correlated with hypoperfusion in the prefrontal and cerebellar cortex, thereby indicating the different mechanisms of cognitive impairments in two types of MSA (Minnerop et al., 2007). The underlying mechanisms of the effect of rTMS on cognitive function may consist of increasing LTP (Thickbroom, 2007), enhancing synaptic function (Shang et al., 2016), increasing hippocampal neurogenesis in the dentate gyrus (Ueyama et al., 2011) and leading to network level changes in brain function (Bangen et al., 2012). High-quality research should be further conducted to clarify whether rTMS can improve cognitive function in MSA patients and elucidate the underlying mechanisms.

Cerebellar tDCS were shown to modulate cerebellar excitability in polar-specific manners, as highlighted by the modulation of CBI. Cerebellar anodal stimulation was reported to improve the excitability of the cerebellar cortex, thereby promoting CBI, while cathodal stimulation could reduce excitability, thereby causing CBI to decrease (Galea et al., 2009). Cerebellar tDCS are suggested to work by polarizing Purkinje cells and alternating activity patterns in the deep cerebellar output nuclei (Galea et al., 2009; Grimaldi et al., 2016). Moreover, anodal cerebellar tDCS was reported to reduce the amplitudes of long-latency stretch reflexes in patients with ataxia by increasing the inhibitory effect exerted by the cerebellar cortex on the cerebellar nuclei (Grimaldi and Manto, 2013).

This systematic review shows the advantages that only peer-reviewed articles were recruited, and that the methodological quality was assessed. However, some limitations remain in this review. First, the total numbers of recruited articles and participants were small. In addition, some articles did not elaborate on the diagnosis of the enrolled subjects, so the authors have no way to distinguish whether the subjects belong to probable MSA or possible MSA. Therefore, the generalization of the conclusion of this review is limited. Second, because of the heterogeneity of interventional protocol and outcome measures among the mentioned articles and an insufficient number of articles in each subgroup, we did not conduct a meta-analysis. Third, gray literature was not searched, and only articles published in English were included, thereby probably causing published bias.

## Conclusions

NIBS can be a useful neurorehabilitation strategy to improve motor and cognitive function in MSA-P and MSA-C patients. However, the effects of other NIBS subtypes on MSA should be investigated more specifically. Further high-quality articles are required to examine the underlying mechanisms of NIBS, to determine the long-term effects of NIBS on motor and cognitive function in MSA patients, as well as to clarify the optimal stimulation protocol (e.g., stimulation site, intensity, duration, and number of sessions).

## Data Availability Statement

The original contributions presented in the study are included in the article/supplementary material, further inquiries can be directed to the corresponding author.

## Author Contributions

The study has been designed by MZ, TH, and QW. Data have been gathered by MZ and TH under the supervision of QW. Data have been analyzed and the manuscript has been drafted by MZ and TH. QW revised the manuscript for important intellectual content. All authors approved the final version of the manuscript.

## Funding

The funding was supported by grants from Rehabilitation Research Project for the disabled of Shanghai (K2018036).

## Conflict of Interest

The authors declare that the research was conducted in the absence of any commercial or financial relationships that could be construed as a potential conflict of interest.

## Publisher's Note

All claims expressed in this article are solely those of the authors and do not necessarily represent those of their affiliated organizations, or those of the publisher, the editors and the reviewers. Any product that may be evaluated in this article, or claim that may be made by its manufacturer, is not guaranteed or endorsed by the publisher.
